# Demonstration of femtosecond X-ray pump X-ray probe diffraction on protein crystals

**DOI:** 10.1063/1.5050618

**Published:** 2018-10-01

**Authors:** Nadia L. Opara, Istvan Mohacsi, Mikako Makita, Daniel Castano-Diez, Ana Diaz, Pavle Juranić, May Marsh, Alke Meents, Christopher J. Milne, Aldo Mozzanica, Celestino Padeste, Valérie Panneels, Marcin Sikorski, Sanghoon Song, Henning Stahlberg, Ismo Vartiainen, Laura Vera, Meitian Wang, Philip R. Willmott, Christian David

**Affiliations:** 1 Paul Scherrer Institut, CH-5232 Villigen-PSI, Switzerland; 2 C-CINA, Biozentrum, University of Basel, CH-4058 Basel, Switzerland; 3 Swiss Nanoscience Institute, CH-4056 Basel, Switzerland; 4 Deutsches Elektronen-Synchrotron DESY, Notkestrasse 85, D-22607 Hamburg, Germany; 5 European XFEL GmbH, Holzkoppel 4, D-22869, Schenefeld, Germany; 6 LCLS, SLAC National Accelerator Laboratory, 2575 Sand Hill Road, Menlo Park, California 94025, USA

## Abstract

The development of X-ray free-electron lasers (XFELs) has opened the possibility to investigate the ultrafast dynamics of biomacromolecules using X-ray diffraction. Whereas an increasing number of structures solved by means of serial femtosecond crystallography at XFELs is available, the effect of radiation damage on protein crystals during ultrafast exposures has remained an open question. We used a split-and-delay line based on diffractive X-ray optics at the Linac Coherent Light Source XFEL to investigate the time dependence of X-ray radiation damage to lysozyme crystals. For these tests, crystals were delivered to the X-ray beam using a fixed-target approach. The presented experiments provide probe signals at eight different delay times between 19 and 213 femtoseconds after a single pump event, thereby covering the time-scales relevant for femtosecond serial crystallography. Even though significant impact on the crystals was observed at long time scales after exposure with a single X-ray pulse, the collected diffraction data did not show significant signal reduction that could be assigned to beam damage on the crystals in the sampled time window and resolution range. This observation is in agreement with estimations of the applied radiation dose, which in our experiment was clearly below the values expected to cause damage on the femtosecond time scale. The experiments presented here demonstrate the feasibility of time-resolved pump-multiprobe X-ray diffraction experiments on protein crystals.

## I. INTRODUCTION

### A. Protein crystallography as one of the X-ray free-electron lasers' (XFELs') applications

Research on biomolecular systems is a rapidly developing application of X-ray free-electron laser (XFEL) facilities.^1^ These ultra-bright X-ray sources provide pulses on the femtosecond timescale with millijoule pulse energies, which allow pump-probe experiments to investigate fast dynamic processes in proteins.^2–8^ In addition to providing feasibility of time-resolved measurements on proteins, the ultrashort pulses of XFELs can overcome the radiation damage limitations encountered when using synchrotron radiation. In this case, the investigation of microcrystals is limited by the fact that radiation damage often prevents the collection of useful diffraction datasets when using very intense, tightly focused X-ray beams. Due to the femtosecond pulse lengths of XFELs, recording of high-quality diffraction patterns from the probed crystals is possible before structural damage sets in. By sequentially collecting diffraction patterns from newly supplied crystals with identical structure and random orientation, it was demonstrated that it is possible to collect diffraction data suitable for solving the molecular structure of proteins.^9^ More recently, there has been a growing interest of structural biology in serial protein crystallography at XFELs facilities, and the method has led to increasing numbers of solved structures.^10–18^

### B. Radiation damage mechanisms in protein crystals

Protein crystals consist mostly of only low-Z atoms and significant amounts of solvent (water).^19,20^ They usually have large unit cells. Destruction of these radiation sensitive samples^21^ is based mostly on photoelectric absorption. Classification of the radiation damage within an irradiated protein crystal can be based on the location within the crystal in which it occurs in. Namely, they are considered global when large displacements of the protein molecules are observed and therefore there is an increase in the mosaicity, the unit cell constants change, and the loss of the resolution occurs. On the point/local scale, the most prominent damage processes include the cleavage of the disulphide bonds (S-S), decarboxylations of the amino acids, or photo-reduction of redox systems. All these phenomena occur on their characteristic time scales.^22,23^

The fastest time-scale of radiation damage processes gives the limit for serial femtosecond crystallography (SFX) and so-called “diffract-and-destroy” data collection mode,^24,25^ where the sample is blasted by the X-ray laser pulse, but only after the scattering process has taken place, which can be registered as diffraction pattern on the detector. Theoretical investigations have been performed in order to quantify the damage effects and to reveal the relevant mechanisms.^26–28^ First simulations reported by Neutze *et al.* on protein lysozyme molecules approximate femtosecond X-ray induced Coulomb explosion to occur on sub-100 fs time scales.^29^

### C. Experimental approaches to radiation damage at XFELs

Experimental approaches have focused on the effects of pulse duration and fluence in SFX and comparison to data obtained at synchrotrons.^30,31^ Chapman *et al.* reported on SFX data on lysozyme crystals collected with 200, 70, and 10 fs pulse lengths.^32^ It was found that the Bragg-peak intensity drops by about one order of magnitude when comparing 200 fs to 70 fs pulses or shorter. These findings indicate that only the photons arriving during the first tens of femtoseconds of an X-ray pulse interact with an intact crystal. Photons arriving later interact with a sample of decaying crystallinity, which contributes less and less to the diffraction peaks and merely adds to the diffuse background. This self-terminating diffraction mechanism^33^ is predicted to greatly help in the collection of SFX data even with pulse length exceeding the time scales of structural damage.

So far, no direct measurements of the dynamics of femtosecond radiation damage in protein crystals have been demonstrated. There are two main obstacles that have impeded such experiments in a classical pump-probe manner. Firstly, it is very difficult to provide an intense X-ray pump beam and an X-ray probe beam onto the same sample location but with a defined delay on the femtosecond scale. Secondly, the repetitive data collection at varying delays would require delivering fresh, undamaged crystals for every shot, under precisely the same Bragg angles.

**FIG. 1.**
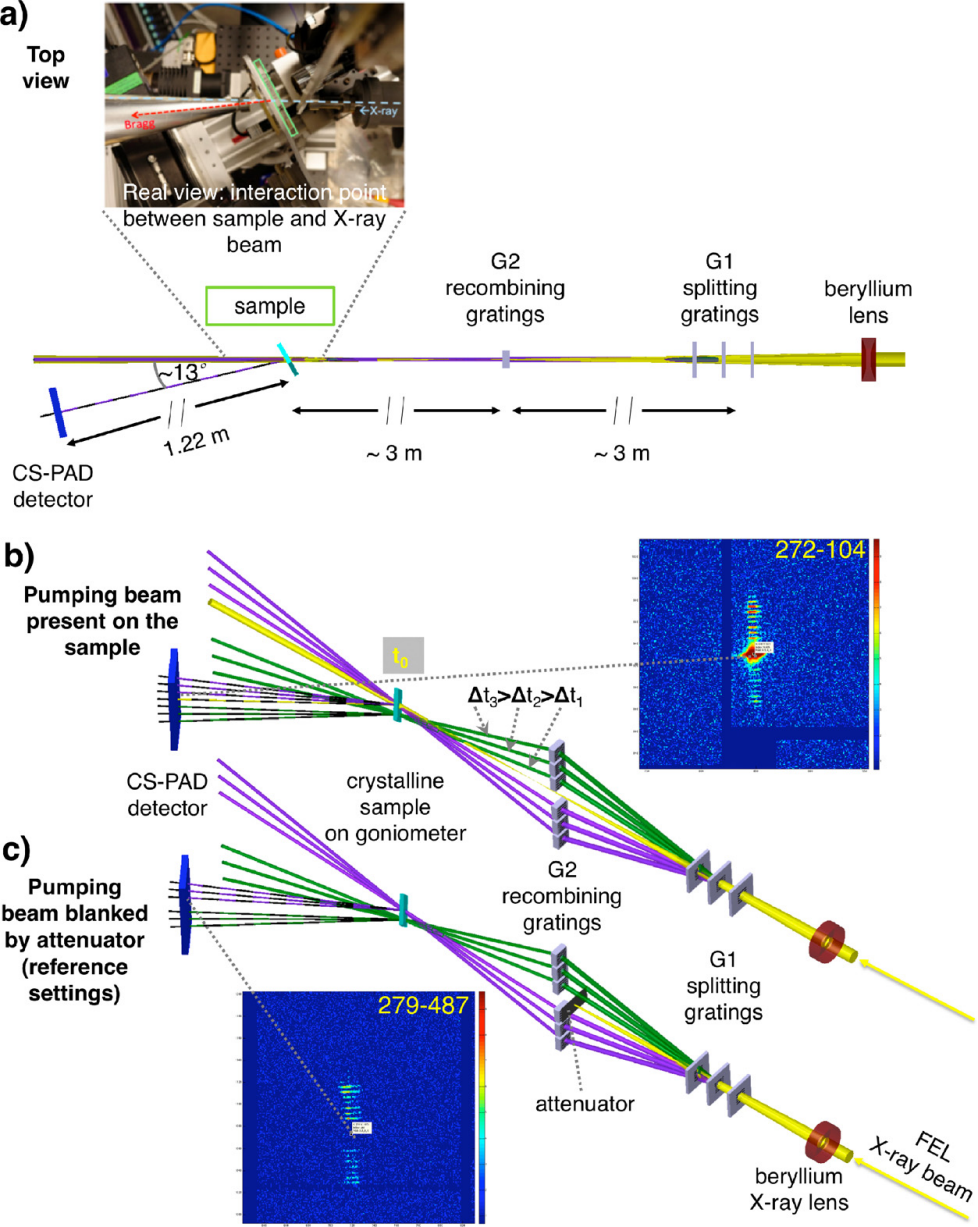
Scheme of the X-ray optics set-up (adapted from Ref. 34), showing the data collection in transmission geometry for protein crystal diffraction. The detector was placed off-axis at an angle of 13° at a 122 cm distance from the sample. (a) Top view, (b) perspective view, pumping beam on (spot size focused down to 40–50 *μ*m), and (c) perspective view, pumping beam blanked. The dashed lines (― ― ―) indicate diffracted beams from the crystals collected ideally on the single tile of the CSPAD. The inset shows only a part of the detector with the signal. The purple probing beams run through the region of the sample hit by the pump beam, while the green probing beams run through a region of the sample not overlapped with the X-ray pump (b).

Application of the pump/multi-probe setup, as reported here, can directly access femtosecond events in crystalline protein samples using X-ray diffraction. Our new approach to investigate the ultrafast X-ray damage processes in protein crystals covers, in this case, a time-scale from tens to up to a few hundred of femtoseconds and presents a proof of concept experiment. The investigated time window is of particular importance to explore the effects of beam damage in protein crystals probed with XFEL radiation with ultrashort pulses. For this purpose, the split-and-delay setup^34^ was adapted for measurements in transmission (Laue) geometry (Fig. 1). Experiments performed with this setup allow for studying the effect of X-ray interaction with biological matter by observation of the measured diffracted signal collected on an integrating pixel array detector. The delayed peaks accompanying the main Bragg reflections from the probed protein crystal reveal the evolution of the Bragg-reflected intensity in time.

## II. EXPERIMENTAL AND COMPUTATIONAL METHODS

### A. Experimental setup and diffraction geometry

For the experiments presented here, the set-up follows the design of an X-ray optical delay line as described by David *et al.*^34^ (Fig. 1). This provides the possibility to measure multiple discrete delayed probes with the same X-ray pumping beam using geometrically defined, jitter-free timing. A set of beryllium lenses was used for focusing/defocusing the direct beam to approximately 40–50 *μ*m (ø) spot size on the crystals, and a set of delayed beams was generated from the main beam by a split-and-delay type of setup. It consists of an arrangement of X-ray diffraction gratings (“G1 gratings”) that splits a multi-keV X-ray pulse into an intense pump pulse and a fan of delayed probe beams, which are redirected by a second set of gratings (“G2 gratings”) to intersect at the target position, where the sample is placed. Due to the longer paths, the probe pulses arrive with precisely defined time delays at slightly different incident angles. Moreover, a second symmetrically arranged set of delayed reference beams arrives at the sample with a spatial offset of 70 *μ*m with respect to the pump beam and irradiates a part of unpumped sample area to serve as reference to the measurement. The obtained delay times (cf. Table I) corresponded directly to the grating pitches and distances. The 4th time delay of 77.7 fs was obtained by using the 2nd diffraction order of the 1st grating.

**TABLE I.** Length of individual time delays.

No.	1	2	3	4	5	6	7	8	9	10	11
Delay time [fs]	**19.4**	**34.0**	**53.8**	**77.7**	**106.3**	**138.1**	**173.0**	**212.2**	**256.5**	**307.0**	**357.3**

After being diffracted from the same crystal plane, the pump, probe, and reference beams reach the detector at slightly different positions (Fig. 3). This allows for registering the time-resolved diffraction signal without the need of an ultra-fast detector. Thus, the femtosecond dynamics of the excited sample can be detected for a single X-ray pump pulse as a streak of delayed peaks around the main Bragg peak originating from the beams of an intensity approximately 10^6^ times fainter than the pump beam.

The experiments were performed at the X-ray coherent scattering (XCS) end-station of the Linac Coherent Light Source (LCLS) XFEL source, operated at a nominal photon energy of 5 keV with a spectral bandwidth of the single shot of about 10–15 eV FWHM^35,36^ and a nominal pulse duration of about 45 fs, meaning that the first probes are arriving at the pumped area while pumping still occurs.

Technical modifications as described below were made to the original design^34^ to provide a better match towards the investigations of protein crystals during the first few hundred femtoseconds after excitation. The setup was reduced in length by almost a factor of two, i.e., to approximately 6.6 m from the G1 gratings to the sample position. The new design was used at slightly increased photon energy of 5 keV and produced 11 delayed beams covering a time window of up to 357 fs with denser sampling instead of the previous 15 beams reaching up to 1200 fs delay.

As typical for protein crystallography experiments, the measurements were performed in transmission (Laue) geometry. The G1 diamond beam-splitting gratings were equally spaced by 16 mm along the beam axis, each mounted on a rotational stage for an alignment performed around the beam axis with assistance of beam spots visualization on the scintillator screen.

To increase the efficiency of the narrowest pitch gratings (8th, 9th, and 10th), they were filled with iridium.^37^ The G2 gratings set, which recombines the split beams and brings them to the sample at the incident angles with up to 5.7 mrad differences, was slightly asymmetric. This resulted in the lateral shift on the sample of the probe and reference parts of the split beam of approximately 70 *μ*m. This set-up is also described in more detail by Makita *et al.*^38^ showing its application for studying dynamics on bismuth single crystals in reflection (Bragg) geometry. These experiments also confirmed the spatial overlap of the pump and probe beams originating from the used X-ray optics setup.

The diffracted patterns from protein crystals were recorded at room temperature on a Cornell-SLAC Pixel Array Detector (CSPAD) 2.3 M, a pixelated detector (pixel size: 110 *μ*m × 110 *μ*m) with an overall size of about 20 cm × 20 cm.^39,40^ It was placed off-axis in the horizontal direction at an angle of approximately 13° from the direct beam and at a distance of 122 cm from the X-ray/sample interaction region, as schematically shown in Fig. 1. A shorter detector distance would not have given enough angular resolution to resolve the delayed beams and would have concentrated too much scattered intensity on too few pixels, potentially causing damage to the detector. On the other hand, placing the detector too far from the sample would have reduced the probability of capturing relevant reflections. The detector position was calibrated using silver behenate (AgC_22_H_43_O_2_), a crystalline powder with 58.380 Å d-spacing,^41^ sandwiched between two pieces of adhesive Kapton tape. The resulting equally spaced arcs of powder diffraction pattern confirmed that the geometry was chosen in a way to cover a part of the solid angle corresponding to the d-values ranging from 8 Å to 15 Å (corresponding to a q-range between 0.4 Å^−1^ and 0.7 Å^−1^) (supplementary material Fig. S1). This covers the reciprocal space where lysozyme crystals yield the most intense Bragg reflections.

### B. Crystalline protein sample delivery

For diffraction data collection, protein crystals of chicken egg-white lysozyme with sizes between 50 and 400 *μ*m were prepared by three different procedures. Two of them used *in situ* growth of the crystals. Crystallization was carried out on arrays of ultrathin silicon nitride membranes in microfabricated silicon chips from manually deposited solutions. For taking measurements, the samples were hermetically sealed with a second chip with silicon nitride windows as described previously in detail.^42^ Alternatively, drops of protein solution were automatically dispensed using a Mosquito^®^ crystallization robot on 25 *μ*m thick cyclic olefin copolymer (COC) bottom of standard-dimension 96-position microtiter well plates.^43^ The procedure was applied as follows: lysozyme protein from chicken egg-white (Sigma-Aldrich L6876‐10G) at a concentration of 50 mg/ml (in 50 mM sodium acetate, pH 4.5) was crystallized by vapor diffusion against the precipitant solution (2M NaCl, 5% poly(ethylene) glycol monomethyl ether 5000, 50 mM sodium acetate pH 4.5, 25% ethylene glycol). A 200 nl drop of lysozyme was dispensed at the bottom of each CrystalDirectTM Plate well (MiTeGen LLC)^44^ together with a 200 nl drop of the precipitant. The crystallization mixture was equilibrated against 500 *μ*l of precipitant. The plate was efficiently sealed using a ClearVue Sheet (Molecular Dimensions) and incubated for one day at room temperature. The sealing cover was replaced immediately before the XFEL measurement by a less watertight but X-ray transparent 25 *μ*m-thick Kapton^®^ foil (Kapton type HN, DuPontTM, USA), attached with 141 *μ*m-thick 96-well adhesive gaskets from Saunders^®^. This approach provided a perfect environment for each crystal to grow to the desired dimensions, until precisely located and probed. A third type of sample was also prepared with the use of the silicon nitride membrane chips. The difference is that the crystals were initially grown in a crystallization plate then fished and transferred by hand between two silicon nitride membranes. Successful data collection is feasible from each kind of tested samples. However, the silicon/silicon nitride chip approach provided the most suitable environment for efficient serial data collection, due to the much lower background level (supplementary material Fig. S2) originating from crystal packaging. This is due to the very thin membranes and limited amount of mother liquor around the crystals, as well as the regular placing of crystals on a small area.

### C. Pump-multiprobe femtosecond data collection

**FIG. 2.**
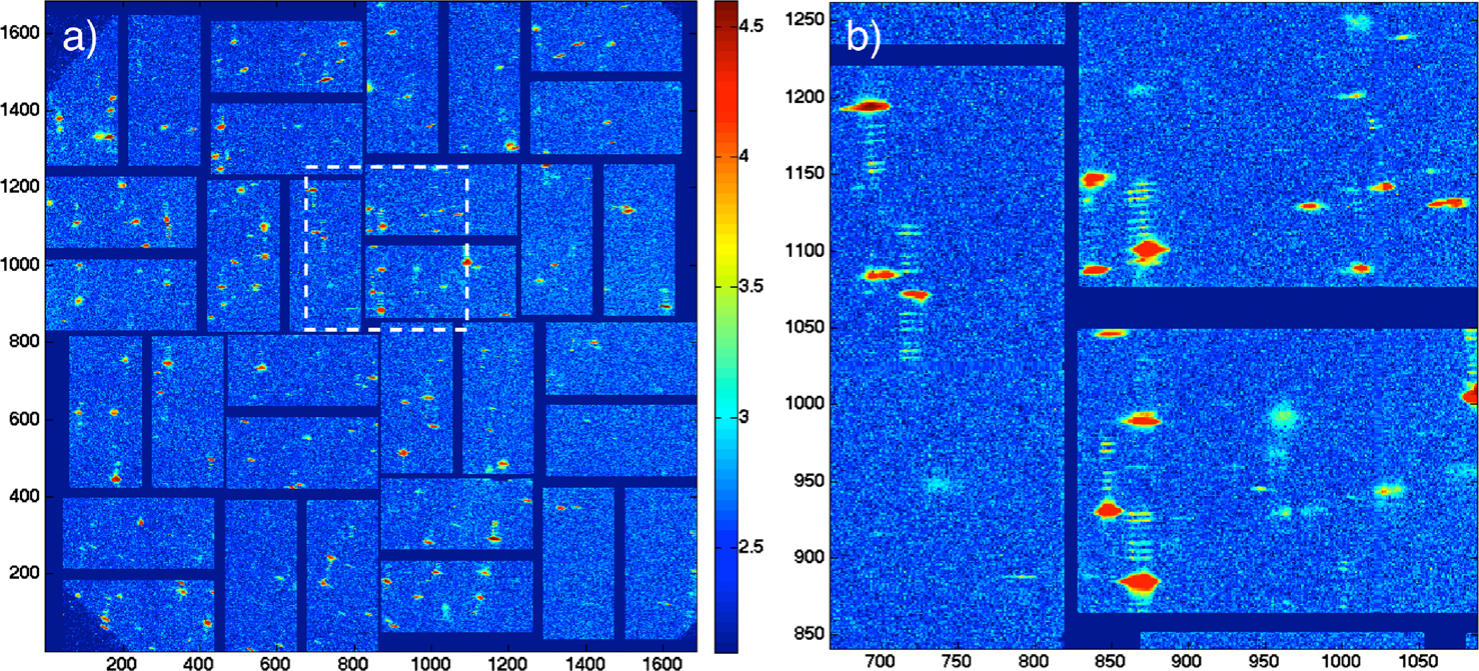
Summed signal of the 289 shots selected for analysis after initial filtering. The diffraction signals originate from the lysozyme crystals grown/deposited on silicon nitride windows. The intensity scale of the images is log_10_(I). (a) Full view of the CSPAD and (b) zoomed view of the marked region.

**FIG. 3.**
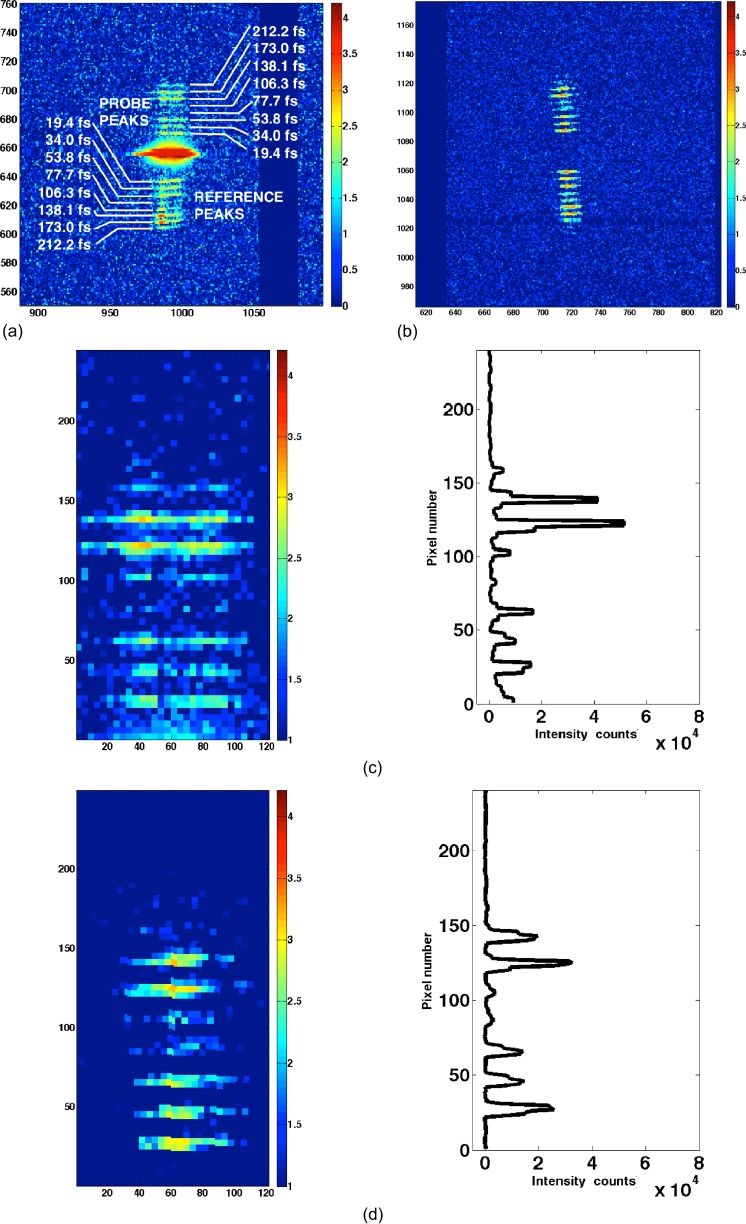

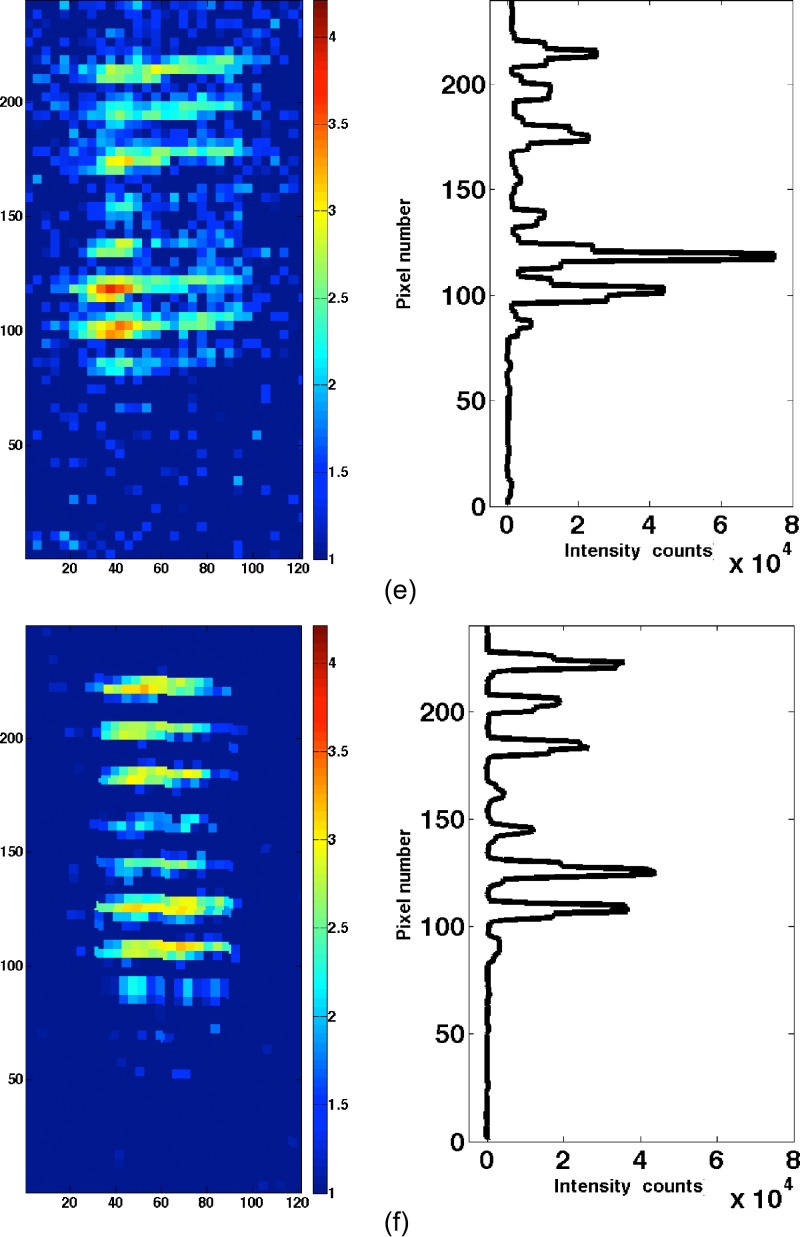
Examples of registered diffraction signals of (a) a pumped shot (Run 275-Shot 136) and (b) an unpumped shot (Run 277-Shot 326). (c)–(f) Regions of interest selected for intensity integration after resizing (images in logarithmic scale log_10_(I), resizing factor: 4, original size: 30 px × 40 px) and delayed peaks' intensity profiles (linear scale representing values of counts registered on the detector after integration in x direction) of measured intensity distribution for all visible delays in a shot. (c) and (d) Probe and (e) and (f) reference part of the signal of (a) and (b), respectively.

Two different types of datasets were recorded, one with the main direct beam at its highest intensity (full XFEL beam) running through the sample (pump) [Fig. 1(a)] and the other with the pump beam blanked out [Fig. 1(b), cf. Figs. 3(a) and 3(d)] to serve as an unpumped reference. A total number of 10 905 shots were collected during the 12 h of an LCLS beamtime shift. The collected images of the exposures were visually inspected and individually selected. This resulted in a set of 289 shots with visible diffraction signals originating from protein crystals supported by the silicon nitride substrates. Figure 2(a) shows the full set of these shots, which have been used after initial filtering for further analysis. In Fig. 2(b), a zoomed view is presented, showing a selection of reflections close to the detector center including some of the characteristic delayed peaks.

Since no rotation of the crystal was possible during the exposure to the X-ray pulses (crystal damaged after each shot) and due to the limited solid angle covered by the detector, the resulting diffraction patterns contain only a single or at maximum two Bragg peaks. Partial signals, meaning that only probe or reference sides were visible next to the Bragg peak, frequently occurred. This is due to the fact that only one series of the delayed beams reached the crystalline sample. The reason for this was either that the size of the protein crystal was smaller than the distance between the probe and the reference area spots (70 *μ*m) or that the location of the crystal on the chip was such that part of the beams missed the crystal.

The images with visible patterns were graded A, B, or C depending on the quality of the signal intensity and then selected for further processing (supplementary material Fig. S3). Individual images with well-visible and separated delayed peaks signals (Grade A shots) of the delays on the probe-side, the reference-side, or on both sides together (“complete” shots [supplementary material Fig. S4(a)]) were further analyzed. They created the following four datasets: probe pumped 60, probe unpumped 29, reference pumped 37, and reference unpumped 33 exposures.

### D. Procedure for calculating line profiles of diffraction intensities

Following the visual inspection and manual determination of the positions of the collected signals, further processing was performed on this limited dataset. Signal profiles were extracted by means of MatLab scripts [Figs. 3(c)–3(f)]. The applied algorithms are able to identify the position of the signal on the basis of manually given input coordinates on the center of the 6th (the most intense out of registered) delay peak. From this, they determine the integrated intensities of the delayed peaks (Fig. 3). Data treatment involved: (i) correction of the angle originating from the position on the detector by rotation, (ii) obtaining line profiles of the signal by summing up the selected region of interest over the width of the signal (Fig. 3), and (iii) subtraction of the background for each peak separately.

## III. RESULTS AND DISCUSSION

### A. Estimation of the dose on the exposed crystals

The relatively large area of the pump beam (40–50 *μ*m, vs Sec. II A) was kept to ensure lateral overlap of both the pump and the probe beams on the crystal. Focusing to a smaller spot size would have increased the local dose at the risk of incomplete beam overlap.

XFEL pulses of approximately 45 fs, at an average of 3.2 mJ energy, at a wavelength of 2.48 Å (5 keV) were used for the pump-probe experiments on protein crystals grown on the solid supports. However, a substantial part of the initial energy of the XFEL pulses was lost on the way to the sample, mainly because of absorption by the optical elements in the X-ray path. About half of the initial intensity was lost by absorption and diffraction by the diamond membranes of the G1 gratings (each 10 *μ*m thick) and a similar fraction was absorbed by the beryllium focusing lenses. Additional photon intensity losses occurred in the 125 *μ*m thick diamond exit window and in the helium-filled flight tubes, which are placed between the G1 gratings and the sample as well as between sample and detector. The out-of-vacuum beam path further contributed to the reduced level of delivered dose. The final X-ray energy arriving at the sample was measured to be approximately 100 *μ*J. For this purpose, a calorimeter has been placed at the sample position with all the optics in the X-ray path (including compound refractive lenses, G1 gratings, direct beam focusing lens, and no aluminum filters inserted).

Taking into account the approximated 5 keV photon energy X-ray transmission through lysozyme crystals with about 45%–98% (for thicknesses of the crystals between 5 and 200 *μ*m), the density of the crystals (≈1.2 g/cm^3^) and the incoming pulse energy of 100 *μ*J result in an estimated dose in the spot area of about 0.1–0.2 MGy.

### B. Diffraction data collection

During the initial alignment runs and final tuning of the experimental set-up, large crystallization plates backed with kapton foil with grown lysozyme crystals (cf. Sec. II B) were probed in “by hand” mode, aligning each crystal individually. This method of collecting diffraction images resulted in a high hit rate but was very time-consuming. Subsequently, the silicon nitride membrane arrays with large lysozyme microcrystals (50–200 *μ*m) were scanned through the XFEL beam and were exposed window by window in an automatic fast scanning manner with a repetition rate of 1 Hz. Due to the quasi-random position, the crystallographic orientation of the crystals in the windows, and placing of shots in a regular pattern, the hit rate was lower. However, the overall yield of diffraction patterns was much higher compared to manual strategy described above due to the significantly increased repetition rate. Even though the sealed sandwiches with regularly placed samples were open to the ambient environment after the first shot, it appears that the dehydration caused by vapor exchange with the normal atmosphere was slow enough that it did not affect the quality of the subsequent shots. Ultimately, diffraction data could be collected from a whole chip (196 wells/windows). During the data collection, all the samples were kept at ambient conditions (RT, normal pressure). Two sets of experiments were run, one with the pump and one without (i.e., delayed peaks only), with the latter being used to evaluate the relative intensity of the delayed peaks and also possibly for calibration purposes.

Due to the small solid angle in the chosen detector geometry, only one or two Bragg peaks could be recorded from a single X-ray pulse. Therefore, the determination of the approximate d-values corresponding to the lysozyme Bragg peaks was possible, but no indexing. The main Bragg peak of the registered signal was in most cases either saturating the detector [supplementary material Fig. S4(a)] or not present in the unpumped shots [supplementary material Fig. S4(b)].

This saturation, visible in the set of the pumped shots resulted in a locally increased background (flare) around the main Bragg peak. The spatial separation of the delayed peaks on the CSPAD proved to be sufficient for the quantification of the intensity of the delayed peaks. The delayed peaks were visible up to the 10th delay (307.0 fs), but the lower intensity delays (9th, 10th, and 11th) were often lost in the noise and did not provide usable data.

Unfortunately, only rarely were both probe and reference sides of the diffraction signal registered from the same crystal (supplementary material Fig. S4). These few acquired complete data frames confirm the possibility to collect simultaneous pump and reference information with our method. Since the statistical relevance of this sparse dataset is questionable, we pursued the alternative approach for data processing that considers delayed peaks from the probe and reference side separately. In future experiments, the yield of the registered diffraction peaks could be increased by using larger crystals in width and length (but not in thickness), and by applying a better (automated) alignment procedure as well as prelocation methods.

Additionally, some shots had to be excluded from further analysis, as they partially fell on the inactive gaps between the detector tiles. High signal rotation and loss of part of the delayed peaks occurred when the signal was diffracted under high angle away from the main X-ray propagation axis resulting in landing at a largely off-centered position on the detector. In such cases, delayed peaks are prone to falling off of the rocking curve, meaning that they do not fully fulfill Bragg's law (supplementary material Fig. S5). However, in favorable conditions (intense Bragg peak, thick region of the crystal), it was also possible to reach intensity values close to the detector saturation level with the delayed peaks and clearly observe the weakest or the longest delay (4th, 9th, and 10th) peaks (supplementary material Fig. S6).

### C. Data analysis

During the manual pre-selection and grading process (cf. supplementary material Fig. S3), a limited set of data packages (cf. Sec. II C, supplementary material Fig. S7) were chosen for further evaluation. They consist of the following numbers of A-graded shots: 60 for probe pumped, 29 probe no pumped, 37 reference pumped, and 33 reference no pumped shots (supplementary material Fig. S7). Following the manual localization of the delayed peaks at the detector readout images, further processing was performed using automated Matlab scripts on these relevant shots. Signals were corrected for small rotations and integrated on the horizontal axis [Figs. 3(b), 3(c), 3(e), and 3(f)]. Peak positions were automatically refined and their total intensity integrated after background subtraction. As no information was available about the absolute Bragg-peak intensity, the intensities of the delayed peaks were normalized by their total intensity (sum of the first 8 delays) as an internal reference.

Moreover, due to the different diffraction efficiencies of each grating, there is a large fluctuation between the characteristic relative intensity (supplementary material Figs. S7 and S8) of the different delay peaks. Factors include: limited amount of available data, differences in protein arrangement, and fluctuations in the X-ray pulse spectrum contribute to the high level of signal scattering within a single time data-point (supplementary material Fig. S8).

The relative intensities of the shots in each dataset were subsequently reduced into an average (single) value per delay (supplementary material Fig. S8). Error bars for each time step were determined separately and represent ±1 standard deviation from the calculated average. The averaged values of the relative intensities are different for each time point, due to the different diffraction efficiencies of the used gratings. The strongest signals are found for the 6th and 7th delays representing delay times of 138.1 and 173 fs. The weakest signals were registered in the 4th delay (77.7 fs), because the second order diffraction of a G1 grating was used. It also showed for the longest (e.g., 8th) delays, as the fabrication of the gratings with the feature spacing, which was close to the technical limits, resulted in low diffraction efficiencies. Delays number 9 and 10 were only rarely visible, and the 11th delay was never detected. These relative intensities follow similar trends in the pumped and unpumped sets for both the probe [supplementary material Fig. S8(a)] and the reference [supplementary material Fig. S8(b)] sides and their ratio shows no clear changes beyond statistical noise. Attempts to divide the experimental intensity values of the “pumped” scenario by the average of “unpumped” (plots not shown) give graphs of approximately constant functions at the level of 1, with relatively large errors for all the data points. Specifically, significant errors were observed for the weak signals (4th and 8th delays), as the applied data treatment strategy attaches more importance to the intense values.

**FIG. 4.**
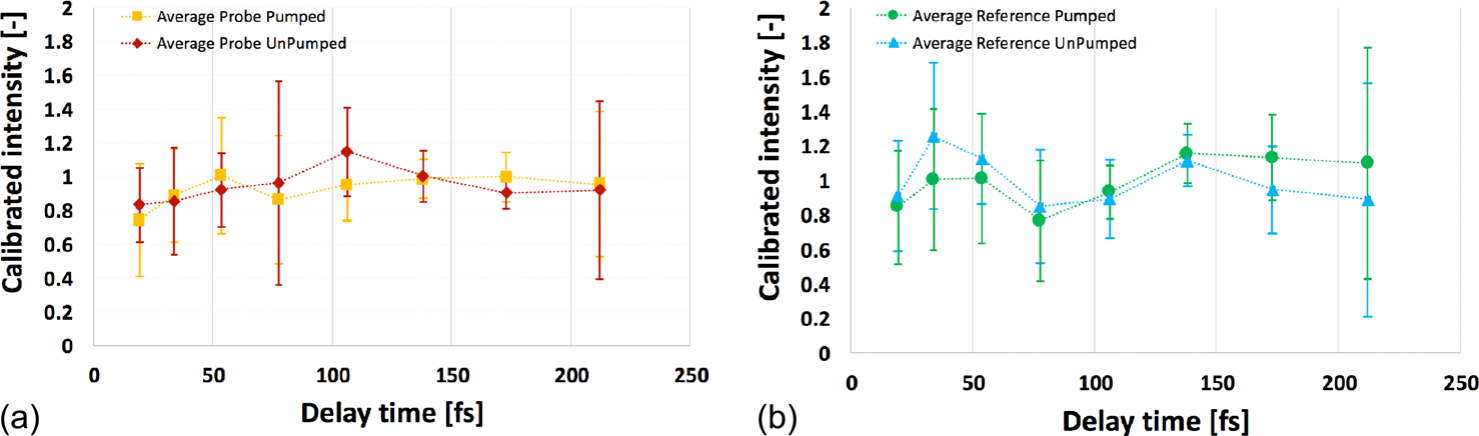
The average intensity based on measurements on protein crystals for individual 8 time points (19.4; 34; 53.8; 77.7; 106.3; 138.1; 173; and 212.2 fs). It has been calibrated with factors obtained from unpumped reference measurements on silicon single crystals. Data shown separately for the parts of the registered signal are: (a) Probe-side pumped/no pump and (b) reference-side pumped/no pump sets. Experimental data are marked by a geometric figure; thin, dotted lines connecting experimental time points are a visual guide and facilitate comparison of the curves. Error bars show ±1 standard deviation of the individual shots from the average truncated to 80% of all the population of the data (sigma value 1.282 applied for shot or single peak intensity value deviating from average).

The obtained results indicate no change in signal that could be assigned to the impact of the pump beam within the studied time window. This can be seen on the intensity plots based on protein crystal diffraction data (Fig. 4) which were calibrated with factors based on unpumped reference measurements on silicon single crystal (cf. supplementary material Table I) collected with the same X-ray optics set-up (Fig. 1), but in reflection geometry. Details about these Si-based results will be published elsewhere. Thus, the data analysis gives no evidence of damage occurring within the studied time window at the applied dose of 0.1–0.2 MGy (cf. Sec. III A).

By applying the data treatment described above, we see no apparent change between the values of the relative calibrated intensities of the delayed peaks for the pumped shots as compared to the corresponding data for the unpumped shots (Fig. 4). Damage of the protein crystal structure on the femtosecond time scale would manifest itself as decaying Bragg reflectivity over time. Chapman *et al.* described this effect previously,^32^ where a strong reduction of the signal intensity was recorded in the resolution-range (15–8 Å), corresponding to the same resolution observed here. The fact that there is no clear decay of the registered signal in our experiment indicates that the dose deposited by the pump beam is too low to cause substantial damage on the probed femtosecond time scale.

**FIG. 5.**
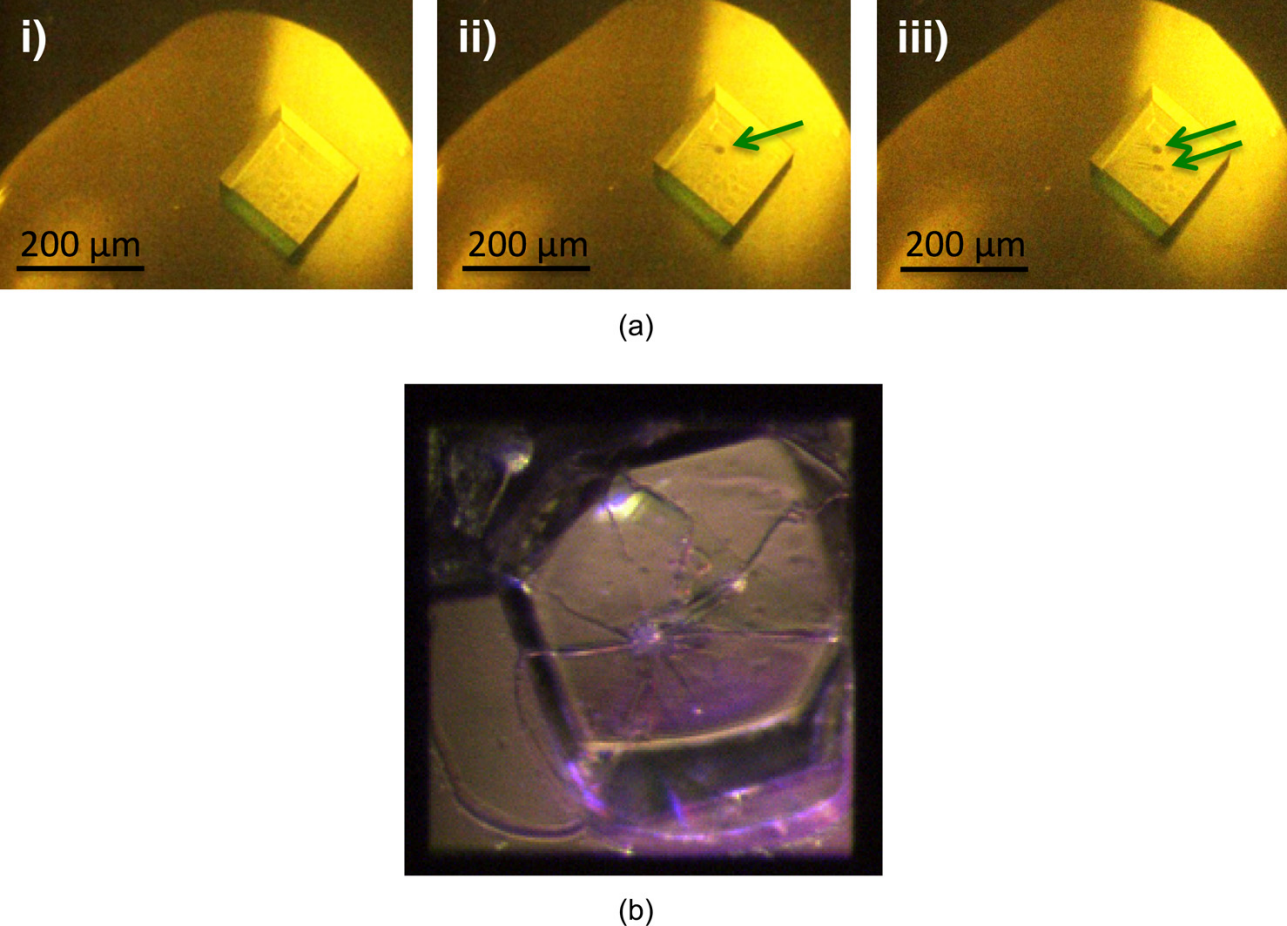
(a) Lysozyme crystal grown on a kapton foil in a plate mounted in the sample position as seen in the mother liquor drop by a video camera: (i) Before exposure to the X-ray beam, (ii) after the first shot (indicated by the arrow), and (iii) after a second shot on the same crystal. (b) Lysozyme crystal grown on the silicon nitride membrane of Chip No. 30, membrane size: 200 *μ*m × 200 *μ*m; cracked after a shot by 5 keV XFEL photons.

However, microscopic inspection of the shot crystals shows evident changes resulting from evaporation of the probed volume of the crystals. The damage in the center of the probed areas is well visible, while the rest of the crystal appears intact. In case of crystals grown on the silicon chips, the silicon nitride was often cracked or broken due to the impact of the pump beam (Fig. 5).

For further optimization of the method for probing protein crystals, the following modifications would be beneficial (i) using prelocation of the crystals before exposures can be used for maximizing hit rate; (ii) running the diffracted beam in vacuum would reduce scattering; (iii) selection of a bigger detector (for higher surface area) would result in covering more of the scattering solid angle, enabling the collection of more than a single Bragg peak per exposure; and (iv) additionally, a second detector placed closer to the sample and therefore covering a larger solid angle would enable collection of a sufficient number of diffraction spots to allow their indexing on the back detector.

## IV. CONCLUSIONS

This study demonstrates the feasibility of diffraction data collection on protein crystals by utilizing an X-ray diffraction grating-based multiple beam split-and-delay line. The applied streaking method can be used to perform time-resolved studies on crystals of macromolecules aiming at a better understanding of the damage processes at femtosecond time scales.

The significant advantages of this approach over conventional pump-probe experiments include (i) no temporal jitter between the pump and the probe pulse, thus the possibility to measure several precisely known delays for each individual pump event.^34^ Moreover, (ii) the ability to control focus/defocus of the pump and probe beams separately allows for an independent adjustment of the pump intensity, and for an optimization of pump and probe spatial overlap. This is not found in any other X-ray probe and X-ray pump methods.^45–48^ The former point is important in the context of protein crystallography, as a conventional pump-probe approach following the response for a single delay only would require renewing the sample for each pump event. Repeating the diffraction experiment for different delays on protein crystals is difficult, as one needs to hit the sample at exactly the same crystal orientation.

The selected sample delivery system based on fixed targets proved to be suitable in providing sufficient amounts of crystalline material at pre-defined positions for serial, automated exposures. Microfabricated silicon nitride membrane chips containing sandwiched crystals were also providing protection of the sample from dehydration. Background scattering from the packaging of protein was much less in the case of the silicon nitride (total thickness: 0.5 *μ*m) in comparison to the thick polyimide (Kapton) foils (cf. supplementary material Fig. S8, Ref. 42). Application of the helium-filled flight tubes further reduced air scattering in the setup.

We were able to record Bragg reflections from lysozyme crystals in the 8–15 Å resolution range that featured clear streaks of delayed reflections. The probed time window here covered 19–213 fs in 8 measurement time points after the initial excitation provided by the main pump beam. Further probe beams with longer delays (9th, 10th, and 11th channel) exist; however, they gave too weak intensities to be taken into account for data analysis. The collected diffraction data give no proof of any radiation damage on this time scale, which is the most relevant time scale for serial femtosecond crystallography. If there were damaging effects of FEL X-rays on protein crystals within the probed time range, they must have been smaller than the error bars of our measurements. These findings are consistent with the approximate values of radiation doses deposited on the crystals in our experiment of 0.1–0.2 MGy (cf. Sec. III A), which are at least one order of magnitude below the dose required for femtosecond damage and also at least three orders of magnitude lower than in the studies described by Chapman *et al.*^32^ Doses applied in our experiment are also relatively low in comparison to doses reported to cause damage in synchrotron measurements that showed no apparent change of the Bragg peak intensity below 1 MGy.^49^

The following improvements can be made for the future attempts of similar experiments, especially addressing the issue of increasing the deposited radiation doses on the probed crystals: (i) Increase of the pulse energy by application of a brighter X-ray source; (ii) increase the transmission of the setup, which was limited to ∼10% in our experiment, e.g., by reducing absorption losses in the diamond exit window of the beamline, or the support membranes of the G1 gratings; and (iii) focusing the photon beam to a smaller spot. Reducing the illuminated area from the 50 *μ*m in diameter as in our experiment to, e.g., 1.5 *μ*m would increase the dose by more than 3 orders of magnitude. Such spot sizes are routinely achieved at XFELs; however, this would put more stringent requirements on alignment to assure sufficient spatial overlap of pump and probe beams.

The presented study is a proof of concept of the single-shot collection of diffraction data from protein crystals probed with several delays after excitation by an XFEL laser pulse. This method, once further optimized, can be applied for studying ultrafast dynamics in proteins.

## SUPPLEMENTARY MATERIAL

See supplementary material for additional figures.
